# Myocardial contractile response to dobutamine in hypoplastic left heart syndrome post-Fontan

**DOI:** 10.1186/1532-429X-16-S1-O104

**Published:** 2014-01-16

**Authors:** James Wong, Kuberan Pushparajah, Adelaide De Vecchi, Gerald F Greil, Tarique Hussain, Reza Razavi

**Affiliations:** 1 Imaging Sciences, Kings College London, London, UK; 2 Paediatric Cardiology, St Thomas' Hospital, London, UK

## Background

The systemic right ventricle (RV) is at increased risk of developing heart failure. Combined MRI-catheterization (XMR) techniques offer a unique opportunity to study this condition. We assess pressure-volume relations in children with hypoplastic left heart syndrome (HLHS) post Fontan with stepwise increments of dobutamine stress.

## Methods

Prospective data from anesthetized patients with HLHS undergoing clinical XMR were analyzed. A fluid filled MR compatible catheter placed in the systemic RV recorded pressures at rest, with dobutamine infusion at 10 mcg/kg/min and at 20 mcg/kg/min. Simultaneous cine short axis stacks of the ventricle were performed. Pressure-volume loops were constructed. End-systolic pressure-volume relationship was derived at each state from a validated maximal pressure (Pmax) estimation method. Ventriculo-arterial coupling (Ees:Ea) was compared to myocardial power and overall global function.

## Results

Five patients with HLHS and exercise intolerance, mean age 7.8 years (range 3.5-11.6 yrs), weight 26 Kg (range 16-46 Kg), time since Fontan completion (mean 3.2 years). No significant tricuspid regurgitation in any patient. Statistical analysis was performed using one-way ANOVA with post-hoc comparisons to assess subgroup variation where p < 0.05. There were significant increases in EF, indexed cardiac output, power and ventricular contractility (Ees) with increases limited to between rest and dobutamine 10 (p < 0.01) with no further significant increase at 20. iEDV fell significantly between rest and dobutamine 20 (p < 0.01). EDP and arterial elastance (Ea) did not change throughout. Ees:Ea increased between rest and dobutamine 10 only. dP/dtmin a measure of rate of relaxation fell with dobutamine 10 only.

## Conclusions

In the systemic RV, load independent indices of contraction in response to dobutamine are similar to those recorded in the healthy left ventricle (LV). The systemic RV at rest works neither at maximal mechanical efficiency (Ees:Ea = 2) nor maximal stroke work efficiency at a given EDV (Ees:Ea = 1). Increased coupling with dobutamine, mediated through increased myocardial contractility, indicates at higher heart rates the RV moves towards improved mechanical efficiency though at higher levels of overall energy expenditure. The relative increase in stroke work (37%) is comparable to data published on the healthy LV. The systemic RV has a limited cardiac reserve. Despite appropriate responses in contractility to low dose dobutamine, the heart is unable to further augment power and cardiac output with administration of dobutamine 20. Indeed, the plateauing of dP/dtmin - a measure of relaxation - indicates that in the presence of tachycardia there is failure to adequately fill the heart. Hence cardiac output and contractility may be limited by a ceiling of maximal flow through the Fontan circuit. This gives us valuable insights into the physiology of children with HLHS post-Fontan.

## Funding

The Division of Imaging Sciences receives support as the Centre of Excellence in Medical Engineering (funded by the Wellcome Trust and EPSRC; grant number WT 088641/Z/09/Z) as well as the BHF Centre of Excellence (British Heart Foundation award RE/08/03). This work was also supported by the European Commission (FP7-ICT-224485:euHeart). The authors acknowledge financial support from the Department of Health via the National Institute for Health Research (NIHR) comprehensive Biomedical Research Centre award to Guy's & St Thomas' NHS Foundation Trust in partnership with King's College London and King's College Hospital NHS Foundation Trust.

**Table 1:** Mean changes in parameters

		Rest	Dobutamine 10 mcg/kg/min	Dobutamine 20 mcg/kg/min	P values
Heart rate	bpm	63	111	137	
Ejection fraction	%	51.4	65.4	65.8	0.01
Power	Watts	14.99	35.41	30.07	0.00007
Indexed Cardiac output	l/min	2.3	4.3	4.5	0.00002
End Diastolic Pressure	mmHg	8.2	6.6	6.2	0.4
dP/dt max	mmHg/ms	0.79	2.53	2.35	0.004
dP/dt min	mmHg/ms	-0.92	-1.81	-1.80	0.002
Ees	mmHg/ml	3.25	5.86	5.45	0.007
Ea	mmHg/ml	2.2	2.7	3.2	0.2
Ees:Ea		1.5	2.2	1.8	0.004
Indexed End diastolic volume	ml/m2	71	59	49	0.001

**Figure 1:**
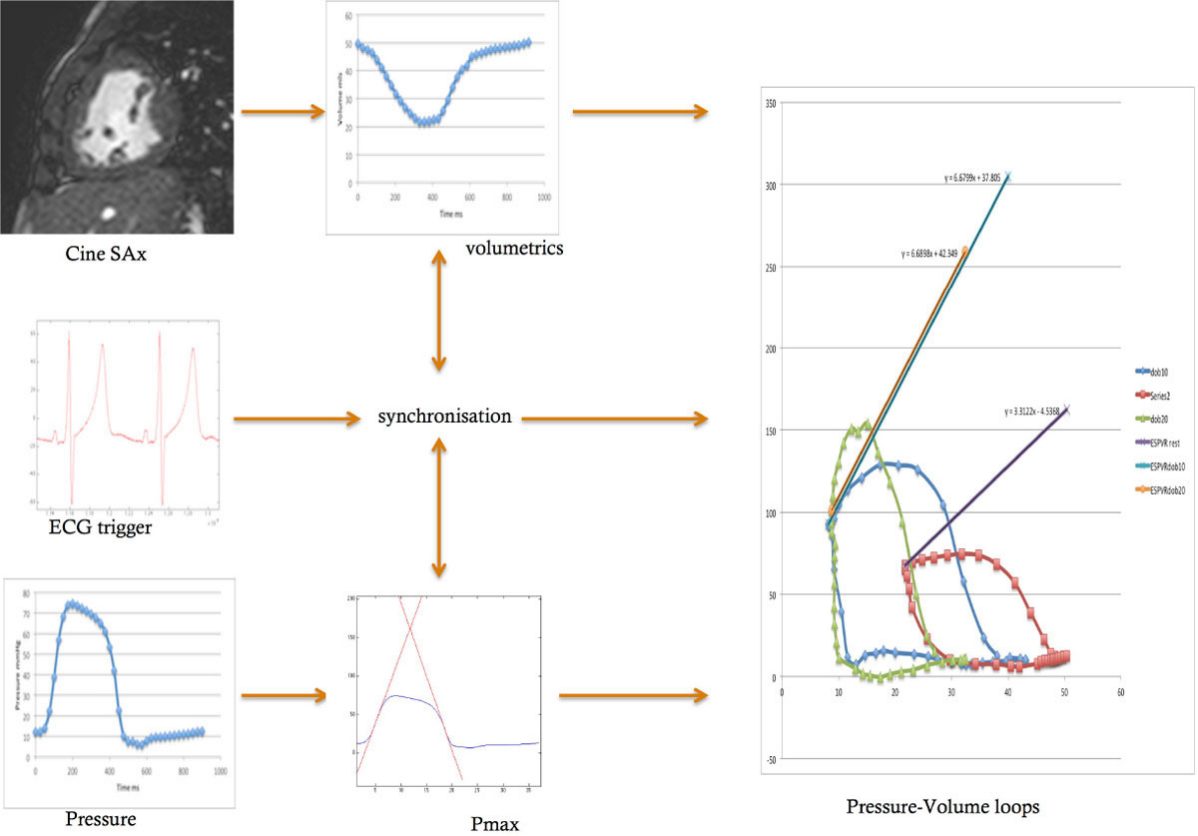
**Data workflow analysis**.

